# Micro-drinking behaviours and consumption of wine in different wine glass sizes: a laboratory study

**DOI:** 10.1186/s40359-017-0183-2

**Published:** 2017-06-12

**Authors:** Z. Zupan, R. Pechey, D. L. Couturier, G. J. Hollands, T. M. Marteau

**Affiliations:** Behaviour and Health Research Unit, Institute of Public Health, University of Cambridge, Cambridge, UK

**Keywords:** Alcohol, Glass size, Tableware size, Drinking behaviour

## Abstract

**Background:**

Tableware size may influence how much food and non-alcoholic drink is consumed. Preliminary evidence of the impact of glass size on purchasing of alcoholic drinks shows an increase in wine sales of almost 10% when the same portion of wine is served in a larger glass. The primary aim of the current study is to test if micro-drinking behaviours act as a mechanism that could underlie this effect, through an increase in drinking rate, sip duration and/or number of sips from a larger glass.

**Methods:**

In a between-subjects experimental design, 166 young women were randomised to drink a 175 ml portion of wine from either a smaller (250 ml) or larger (370 ml) wine glass. Primary outcomes were three micro-drinking behaviours, assessed observationally using video recordings: drinking rate, sip number and sip duration. Other possible mechanisms examined were satisfaction with the perceived amount of wine served and pleasure of the drinking experience, assessed using self-report measures.

**Results:**

Wine drunk from the larger, compared with the smaller glass, was consumed more slowly and with shorter sip duration, counter to the hypothesised direction of effect. No differences were observed in any of the other outcome measures.

**Conclusions:**

These findings provide no support for the hypothesised mechanisms by which serving wine in larger wine glasses increases consumption. While micro-drinking behaviours may still prove to be a mechanism explaining consumption from different glass sizes, cross-validation of these results in a more naturalistic setting is needed.

## Background

Excessive alcohol consumption is estimated to be the fifth leading cause of death and disability [1]. Price, availability, and marketing are key to effective alcohol control policies [2]. Identifying further ways to reduce consumption could usefully contribute to improving population health.

A recent Cochrane review has shown that the size of tableware influences consumption of food and non-alcoholic beverages, with larger sizes leading to greater consumption [3]. However, no studies were found that examined the influence of tableware on consumption of alcoholic beverages. In an initial field study, we found that serving wine in larger glasses, compared to smaller glasses, increased sales by almost 10% [4]. The current study examines micro-drinking behaviours as a potential mechanism for this effect. Other possible mechanisms, including satisfaction with the quantity of the wine served as well as the pleasure of drinking from larger wine glasses, are also examined.

### Micro-drinking behaviours

The mechanisms underlying increased alcohol consumption have rarely been studied. Most evidence for mechanisms underpinning consumption behaviour comes from literature on food, and to a lesser extent, non-alcoholic beverage consumption. Eating rate, bite size, chewing rate, number of sips and sip size have shown to be mechanisms which contribute to the volume of food and non-alcoholic beverage intake [5–11]. To our knowledge, just one study has experimentally compared micro-drinking behaviours (drinking rate, number of sips, and sip duration) for alcoholic beverages served in different glasses. In this study, students were randomised to be served beer in straight or curved glasses. Those who drank from a curved beer glass had a faster drinking rate and took longer and more frequent sips. The authors hypothesised that this was due to drinkers titrating their consumption rate based on the perceived amount of drink in their glass, which was misjudged to a greater extent when served in a curved glass [12].

Wine served in larger glasses is likely to be perceived as less in quantity than a similar amount served in a smaller glass [13]. Such differences in perception may increase consumption of wine served in larger glasses in several different ways. Glasses perceived to contain a lesser amount of an alcoholic beverage due to their shape, may be drunk more rapidly [12]. However, whether glasses perceived to contain a lesser amount of beverage due to their size [12] are also drunk more rapidly has not yet been examined. The primary hypothesis to be tested in the current study is that the same amount of wine is drunk more quickly when served in a larger, compared to a smaller, glass. Related mechanisms found to influence greater consumption of liquids are number of sips [10], and sip size and duration [9, 11, 12]. Thus, micro-drinking behaviours that contribute to rate of consumption, including number of sips and sip duration, may also impact on the amount of wine consumed when served in different glass sizes.

### Other possible mechanisms

Other mechanisms may mediate micro-drinking behaviours or independently affect consumption of alcohol when served in different sized glasses. These include, first, satisfaction with the quantity of the wine served, and second, the pleasure associated with the drinking experience.

Satisfaction with the quantity of the wine may operate through the “unit bias heuristic” [14]. The unit bias heuristic postulates that people consume in “units” (e.g., one plate or one glass), perceiving it as an appropriate amount to consume if it is above a certain “minimum” amount. Since the same volume of wine in a larger glass is hypothesised to be judged as less than when presented in a smaller glass [13], this may result in it being perceived as less than an appropriate “unit”, leading to increased consumption in order to reach a perceived unit threshold. Dissatisfaction with the perceived portion size in a larger glass may therefore increase consumption in order to compensate for this.

The glasses that are used can influence the pleasure of drinking alcohol [15]. This may increase the amount that is consumed on any one drinking occasion. First, people express a preference for drinking from more elongated containers, with higher containers being perceived as more elongated [16]. Since a larger glass is higher and therefore more elongated, this may enhance drinking pleasure from a larger, in comparison to a smaller, glass. Second, research on food suggests that small portions are more enjoyable [17, 18]. Given that a larger wine glass leads to a perceived smaller portion [13], drinking from a larger wine glass may increase pleasure and in turn consumption.

### The present study

Preliminary evidence from a field study suggests that wine sales may be greater when wine is served in a larger glass [4]. The current laboratory-based study examines several possible mechanisms for this effect. The primary hypothesised mechanism is that micro-drinking behaviours change when consuming a fixed portion of wine in larger compared with smaller glass sizes. Specifically, we hypothesise the following:

1. The same portion of wine served in a larger compared with a smaller glass is consumed more rapidly (Hypothesis 1). We will also explore whether any difference in speed of consumption could be a result of i) a greater number of sips and ii) longer sip duration.
2. Serving a fixed portion of wine in a larger compared with a smaller glass lowers satisfaction with the amount (Hypothesis 2).
3. Wine served in a larger glass leads to a more pleasurable drinking experience (Hypothesis 3).

Since the research underpinning Hypotheses 2 and 3 is scant and indirect, the current study should be considered exploratory. By examining relatively broad mechanisms in this study, we may highlight those dimensions where further exploration could be most beneficial. For instance, if larger glasses lead to greater pleasure when drinking, this could be the result of glass size altering the smell or taste of the wine. Similarly, larger glass sizes may differentially impact the physical ability to take a larger sip. If so, this is likely to be reflected in micro-drinking behaviour variables such as drinking rate or sip duration.

Finally, we will examine whether micro-drinking behaviours, as well as satisfaction with the amount and the pleasure of the drinking experience, are associated with the desire to drink more. Desire to drink more will serve as a proxy for assessing further consumption. Perceived intoxication will serve as a proxy for the perceived amount of wine consumed from a larger and a smaller glass. For instance, if participants who drink from a smaller glass perceive that it contains a greater amount of wine than those who drink from a larger glass, they might also perceive having a greater level of intoxication.

## Methods

### Design

The study used a between-subjects design, with participants randomised to one of two groups to receive 175 ml of wine served in one of two wine glass sizes: (a) smaller (250 ml), (b) larger (370 ml).

### Participants

Participants comprised of 166 female students (age *M* = 22.93; *SD* = 3.52, range 18–42) who drank red wine, were at least 18 years of age, were not currently pregnant or taking any medication that interacts with alcohol, and who had not consumed alcohol in the 12 h prior to the study. The study included only women to minimise gender differences in average sip duration [10]. The study was powered to test the first hypothesis assessing drinking rate, based on effect sizes from a previous study [5]. Power analysis indicated that 160 participants were needed to detect a medium sized effect (d = 0.5) in a two-tailed test with α = 0.05 and power of 0.85.

### Materials and measures

#### Wine glasses

**Fig. 1:**
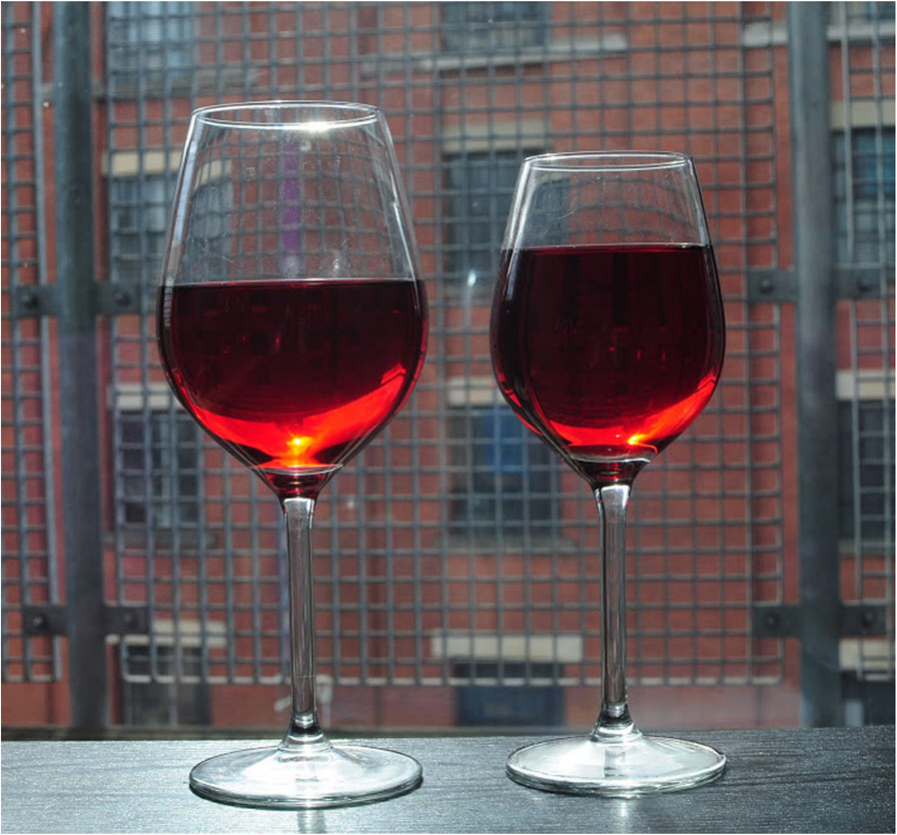
Large 370 ml (*left*) and smaller 250 ml (*right*) wine glasses filled with 175 ml of wine

The larger wine glass was 370 ml in volume and the smaller wine glass was 250 ml in volume. The wine glasses were Royal Leerdam Fortius glasses differing only in their capacity. They were the same as those used in a previous field study documenting higher sales when wine was served in the larger of the two glasses, compared to a 300 ml glass of the same design [4]. The glasses used in the study are shown in Fig. 1.

#### Measures

##### Micro-drinking behaviours

The experimental sessions were recorded using a Raspberry-Pi camera module. The video recordings were coded using a custom-written program in Python (v.2.7), with a researcher pressing a button when the wine touched participants’ lips – indicating sip initiation, and pressing the button again when the wine left participants’ lips – indicating sip end. A second coder, blind to the study hypotheses, independently coded 20% of the videos selected at random to assess coding reliability (presented in the Results section). Variables derived from the video recordings included total time taken to consume the wine, number of sips, and average sip duration.

##### Satisfaction with perceived amount of wine

This was assessed in two parts: firstly, by exploring perceptions of the amount of wine served, and secondly, examining participants’ satisfaction with the perceived amount of wine.

##### Perceived amount of wine

Two questions, rated on a seven-point rating scale ranging from 1 (Much less) to 7 (Much more) asked: “How does the amount of wine in the glass you just drank compare to a typical glass of wine you would drink at home?” and “How does the amount of wine in the glass you just drank compare to a typical glass of wine you would drink at a pub or restaurant?”. The baseline of the typical wine portions participants consumed was established by the following question: “What size would your typical glass in a pub or restaurant be?”. Participants could answer by indicating small (=1), medium (=2), or large (=3).

##### Satisfaction with perceived amount of wine

Five attributes of the given amount of wine (Plentiful, Generous, Inadequate, Unsatisfactory, Disappointing) were each rated using seven-point scales, ranging from 1 (Strongly agree) to 7 (Strongly disagree). The latter three attributes were reverse-coded prior to the analysis. A composite score (‘Satisfaction’) combining these attributes was formed (Cronbach’s α = 0.71).

##### Pleasure

The pleasure of the drinking experience was assessed by rating the experience of drinking the wine on five attributes using seven-point rating scales: Pleasurable, Enjoyable, Disagreeable, Unpleasant, Distasteful. The latter three dimensions were reverse-coded prior to the analysis. A composite score was developed to reflect this variable (Cronbach’s α = 0.95).

##### Desire to drink more

Desire to drink more was assessed by indicating agreement on a seven point scale ranging from 1 (Strongly agree) to 7 (Strongly disagree) for the following statements: “I wish I had another glass of wine right now”, “I don’t want any more wine right now”, “If I were offered another glass of wine right now, I would drink it”, “If I were in a pub or bar, I would buy another glass of wine right now”, “ If I had the chance, I would not have any more wine right now”. Appropriate items were reverse-coded prior to the analysis. Scale reliability assessed by Cronbach’s α was 0.78.

##### Perceived intoxication

Perceived intoxication was assessed with a single item: “I feel drunk at the moment”. Participants responded by indicating agreement with the statement on a scale ranging from 1 (Strongly agree) to 7 (Strongly disagree).

##### Alcohol use

The Alcohol Use Disorders Identification Test (AUDIT [19]), a 10-item measure, was used to assess the quantity and frequency of alcohol use and harmful drinking behaviour. Scores of 0–7 are considered low-risk, scores of 8–14 are considered hazardous and scores of 15 or over are considered harmful.

##### Subjective craving

The Alcohol Urge Questionnaire (AUQ [20]), an 8-item measure, was used to assess current craving for alcohol. The AUQ scores were used as a baseline measure, to ensure that any differences in the outcome measures were not due to urges to consume alcohol.

##### Filler task

The nature of the study was disguised by administering a computerised version of the Trail Making Test as a filler task [21]. The Trail Making Test assesses cognitive processing, visual attention and executive functioning [22–24]. It consists of connecting 25 circles distributed over a computer screen according to set rules (sequentially connecting numbers or alternating between letters and numbers). The dependent variable is the total time required to complete the task. There were two practice trials and two search trials. The results of the filler task were not analysed.

### Procedure

Participants were recruited from the University of Cambridge and from Anglia Ruskin University via mailing lists, poster advertisements and word-of-mouth. They received £10 payment for participation. Participants completed the sessions individually in a quiet laboratory between 12:00 and 21:00 h during weekdays. To avoid participants’ consumption being influenced by awareness of the study hypotheses, the study was presented to them as investigating the effects of limited amounts of alcohol on cognitive performance. After giving consent, eligibility to participate in the study was confirmed by a breathalyser check, to ensure that participants had refrained from consuming alcohol in the preceding 12 h. Participants then completed the AUDIT and AUQ measures. Participants were randomised to either the smaller (250 ml) or larger (370 ml) wine glass condition using a computer generated randomisation schedule with the constraint of having an equal number of participants per group. A portion of 175 ml Cuvée des Vignons Beaujolais red wine (12% alcohol by volume) was measured out by filling a 175 ml pub thimble to the brim, and then poured into the wine glass allocated by randomisation, immediately prior to each experimental session. Wine bottles were secured with a vacuum-pump to minimise oxidation of the wine between experimental sessions. When participants had completed the baseline measures, the experimenter switched on the hidden camera from a remote laptop, and returned to the lab with a glass of red wine. Participants were told to drink the wine at their own pace while watching a nature documentary (“The Story of Earth” National Geographic 2011). The experimenter then left the room and returned when the participants had indicated by ringing a bell that they had finished their glass of wine. If participants did not finish drinking the wine after 30 min, the experimenter returned to ask if everything was alright. If participants were still drinking, the experimenter left and returned to end the session either when the participant indicated that they had the wine finished or after an additional 15 min, whichever was sooner. After the drinking session, participants were given the questionnaires to complete, followed by the filler task. Finally, participants were asked what they thought the aim of the study was. Participants were blind to the study aims and were fully debriefed about the purpose of the study via email at the end of the study, i.e., when the last participant had completed the study.

### Data analysis

Preliminary analyses included examining differences between groups between-group mean differences for effects of glass size on outcome variables using non-parametric bootstraps in R: (i) Total drinking time (ii) Satisfaction, and (iii) Pleasure, and (iv) Desire to drink more, as well as other aspects of drinking behaviour that may contribute to drinking time, i.e.: a. number of sips and b. sip duration. We also tested between-group mean differences for effects of glass size on perceived intoxication as a proxy for how much people believe they had consumed. Regression analyses were conducted to test for “proof of concept”; namely that desire to drink more is predicted by the measures listed in (i) to (iii) above.

Sensitivity analyses were conducted by removing any participants who had not drunk all the wine they had been served or who indicated they were aware of the study aims.

## Results

### Participant characteristics

Descriptive statistics including age, AUQ and AUDIT scores per randomised group are presented in Table 1. There were no differences between groups in age and baseline drinking variables, as assessed by non-parametric bootstrap (25000 replications, α = 0.05 per test), indicating effective randomisation.

**Table 1:** Descriptive statistics of baseline, drinking, and questionnaire variables as a function of glass size

	Larger glass		Smaller glass		*p*
	*M*	*SD*	*M*	*SD*	
Age	23.33	3.88	22.53	3.09	.135
AUQ	18.15	6.28	16.53	5.20	.067
AUDIT	7.30	3.92	6.87	4.03	.496
Number of sips	21.02	8.38	23.57	13.23	.261
Mean sip duration (seconds)	1.46	.48	1.66	.80	.045^*^
Total time (seconds)	1158.42	517.19	983.91	489.34	.024^*^
Amount compared to home	4.43	1.05	4.37	1.89	.745
Amount compared to pub/restaurant	1.87	.68	1.89	.66	.817
Satisfaction	28.92	3.98	29.05	4.97	.915
Pleasure	25.84	6.69	27.08	5.99	.207
Desire to drink more	15.63	7.24	15.53	7.14	.926
Perception of intoxication	2.94	1.45	2.91	1.48	.935

*p < .05, ** *p* < .01, *** *p* < .001

Asterisks indicate significant differences (non-parametric bootstrap, 25000 replications, α = 0.05 for each test)

### Primary outcomes

Descriptive statistics regarding the primary variables are presented in Table 1 and their inter-correlations in Table 2.

**Table 2:** Inter-correlations between baseline, micro-drinking behaviour, and questionnaire variables

	1	2	3	4	5	6	7	8	9	10	11	12	13
1.Glass Size	-												
2. Avg sip duration	.19	-											
3. No. of sips	.11	-.19^*^	-										
4. Total time	-.21	- .23^**^	.35^***^	-									
5. Age	-.14	-.01	-.03	.09	-								
6. AUQ	-.18	-.02	-.01	.03	.06	-							
7. AUDIT	-.07	-.07	-.06	0	-.34^***^	.3^***^	-						
8. Pleasure	.12	-.08	0	.07	.02	.25^***^	.20^**^	-					
9. Desire	-.01	-.05	-.08	-.10	-.08	.44^***^	.49^***^	.47^***^	-				
10.													
Satisfaction	.01	-.05	-.01	.09	.28^***^	-.09	-.19^*^	.19^*^	-.18^*^	-			
11.													
Intoxication	-.01	-.06	.02	.23^**^	.15^*^	-.01	-.09	-.18^*^	-.31^***^	.21^**^	-		
12.Amount at home	-.03	.01	.10	.10	.16^*^	-.04	-.24^**^	-.24^**^	-.35^***^	.20^*^	.27^***^	-	
13.Amount in bar	.02	.05	-.06	-.10	-.13	.20^*^	.50^***^	.19^*^	.45^***^	-.21^**^	-.23^**^	-.50^***^	-
14. Size in a bar	.09	-.11	.02	.17^*^	.29^***^	-.13	-.29^***^	- .11	-.39^***^	.65^***^	.30^***^	.37^***^	-.36^***^

*p < .05, ** *p* < .01, *** *p* < .001

#### Hypothesis 1

Micro-drinking behaviours were analysed by means of non-parametric bootstrap analysis (25000 replications, α = 0.05 for each test; see Table 1). There were significant differences between the larger vs. smaller glass groups in means for total time taken, *p* < .05, with participants drinking more slowly when wine was served in a larger glass.

Differences between the large and small glass groups also emerged in average sip duration, *p*s < .05, with participants taking shorter sips when wine was served in a larger glass. There were no differences with regards to number of sips between the two groups, *p* > .05. Overall, the results did not support the hypothesis that wine is drunk faster when served in a larger glass.

#### Hypothesis 2

The percentage of participants randomised to the larger glass condition that would typically order a small, medium, or large portion of wine was 30.1%, 53.0%, and 16.19%, respectively. Similarly, the percentage of participants randomised to the smaller glass condition that typically take a small, medium or large portion of wine was 27.7%, 55.4%, and 16.9%, respectively. The patterns of participants’ typical wine portion sizes in a pub or restaurant did not differ between groups, χ^2^(2) = .128, *p* > .05.

Differences in satisfaction with the perceived amount were not significant when analysed by non-parametric bootstrap (25000 replications, α = 0.05 for each test), *p* < .05. There were also no differences between the two groups with regards to perceptions of the amount of wine served as assessed by non-parametric bootstrap (25000 replications, α = 0.05 for each test), both *p*s < .05. These results provide no support for Hypothesis 2.

#### Hypothesis 3

Non-parametric bootstrap (25000 replications, α = 0.05 for each test), showed no differences in pleasure, providing no support for the hypothesis that drinking from a larger glass elicits a more pleasurable drinking experience.

### Secondary outcomes

Classic and robust multivariate regression estimates (presented in Table 3) were used to analyse the effects of total drinking time, pleasure, and satisfaction on the desire to drink further. Speed of consumption and pleasure predicted the desire to drink further in the hypothesised direction: the faster the drinking and the more pleasurable the drinking experience, the higher the desire to drink further. There was no statistically significant effect of satisfaction with the amount served on the desire to drink further.

**Table 3:** Classic and robust multivariate regression parameter estimates when analysing the effect of total drinking time, pleasure and satisfaction on the desire to drink more

Classic regression					Robust regression			
	Estimate	Std. Error	*t*	*p*	Estimate	Std. Error	*t*	*p*
Intercept	15.58	.48	38.03	<.001^***^	15.51	.43	36.38	<.001^***^
Total time	−1.05	.41	−2.55	<.05^*^	−1.21	.38	−3.23	<.001^***^
Pleasure	2.60	.45	5.83	<.001^***^	2.79	.45	6.21	<.001^***^
Satisfaction	-.79	.43	−1.82	.071	-.83	.49	−1.68	.095
Age	.19	.44	.44	.663	.05	.43	.11	.911
AUQ	2.16	.45	4.84	<.001^***^	2.31	.47	4.93	<.001^***^
AUDIT	2.07	.46	4.50	<.001^***^	1.93	.56	3.46	<.001^***^

*p < .05, ** *p* < .01, *** *p* < .001

### Reliability check

The ratings of the two independent raters were positively correlated - single measures intra-class correlation for total time was (32) = .99, *p* < .001, and (32) = .99, *p* < .001, (32) = .93, *p* < .001, for number of sips and sip duration, respectively. This indicated a high level of inter-rater reliability.

### Sensitivity checks

Three participants did not finish their wine. The quantities remaining were small and consisted of 3 ml, 6 ml, and 20 ml, respectively. We considered the total time measures of participants with left-over wine as right-censored and compared parameters of gamma regression parameter estimates (models not reported here) and obtained similar results. An additional sensitivity check for participants who correctly guessed the aims of the study (*N* = 12) was conducted and similar results were obtained. Finally, comparison of the between-group location parameters of the distribution of the outcomes of interest by means of Wilcoxon’s tests lead to the same conclusions.

## Discussion

This study examined micro-drinking behaviours (drinking rate, number of sips, and sip duration) as a postulated mechanism for increased consumption of wine when served in a larger glass [14]. Other possible mechanisms, including satisfaction with perceived amount and the pleasure of the drinking experience, were also examined. The results of this study provided no support for any of the hypothesised mechanisms as factors underlying the effects of glass size on consumption, with the only difference in drinking rate being in the opposite direction to that predicted. Drinking rate and pleasure of drinking experience were associated with overall desire to drink further, compatible with two of the three study hypotheses, suggesting that these may prove to be mechanisms influencing increased wine consumption. However, this was not demonstrated between glass size conditions in the current study setting.

There are two sets of possible explanations for the absence of any support for the study hypotheses concerning the mechanisms by which larger glasses increase consumption in a bar. The first set concerns the ecological validity of the laboratory setting for understanding an effect observed in a bar. Consumption of alcohol is influenced by context, with several studies showing that the amount of alcohol consumed in lab settings is generally lower than that consumed in a bar [25–28]. Certain factors, however, have consistently been found to affect drinking behaviour and consumption in both laboratory and field settings, such as paying for drinks, presence of heavy drinking peers, and instructions regarding alcohol content over actual alcohol content [25, 26, 29]. The social environment appears to be of particular importance. Consumption is higher in the presence of heavy drinking peers [29, 30], and social drinkers tend to imitate the number and sip sizes of their drinking partners [31, 32], suggesting that a social environment moderates increased consumption. In the absence of this environment, as in the current study where participants were drinking alone, consumption behaviour may be altered.

A second set of possible explanations for the study’s null findings is that the investigated mechanisms do not relate to alcohol consumption. However, this is inconsistent with our finding that overall drinking rate and pleasure of drinking experience predict the desire to drink further. This supports the ‘proof of concept’ hypothesis and suggests that the measures used in this study can be useful for investigating alcohol consumption behaviour in future research.

### Strengths and limitations

This study is, to our knowledge, the first to investigate the influence of micro-drinking behaviours on wine consumption. While much research has focused on food and portion size, this study investigates the relatively neglected area of alcohol consumption behaviour and the environmental cues such as glass design that can influence it. The use of experimental methods provides the first objective exploration of factors that could be driving increased alcohol consumption from larger glasses.

The limitations of the study include the lack of a consumption-based outcome. While desire to drink more provides a proxy for consumption, a true consumption variable would offer stronger evidence of the mechanisms examined in the current study. A further limitation is that although the laboratory setting allowed for a high degree of control, it may not reflect drinking behaviour in more natural settings, as discussed above. Finally, the study sample differs between the current study and the field study in which the glass size effect was observed [4]. We examined this effect with female university students to reduce gender-related heterogeneity of sip duration. Those drinking in the field study bar would have included men and a wider age range; demographic characteristics that may be associated with drinking behaviours different to those observed in young women in the current study.

### Implications for future research

The current study provides a first step towards understanding the mechanisms by which glass size impacts on alcohol consumption. Future studies will need to ascertain the role of contextual factors that may interact with environmental cues which contribute to alcohol consumption. This can be accomplished by either conducting a field experiment in a bar setting or an experiment in a laboratory setting with added contextual richness (e.g., a bar lab) to determine the external validity of the present results. Together, this could be used to optimise glass design to reduce alcohol consumption at a large scale.

## Conclusions

Examining how a fixed volume wine is consumed from different sized wine glasses in a laboratory setting provided no evidence to support the three study hypotheses. Cross-validation of the present results in a field setting or a bar lab is needed, to exclude the explanations that the present results are an artefact of a laboratory context or inherent to the demographic characteristics of the study sample. Thus, micro-drinking behaviours may still be a promising candidate for a mechanism that can explain consumption from different sized wine glasses, if explored in a naturalistic and ecologically valid setting. Elucidating the mechanisms that underlie modifiable environmental cues remains an important goal for developing interventions that have the potential to inform policies that aim to reduce alcohol consumption at population level**.**
